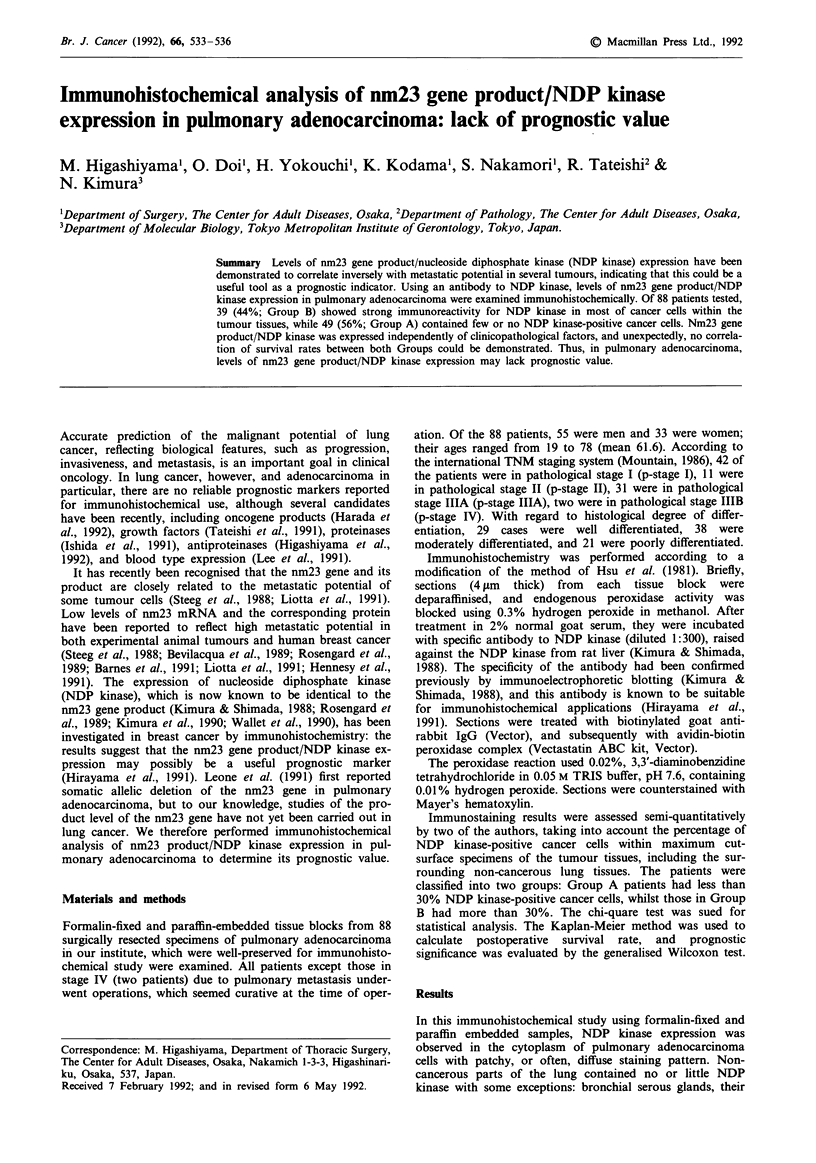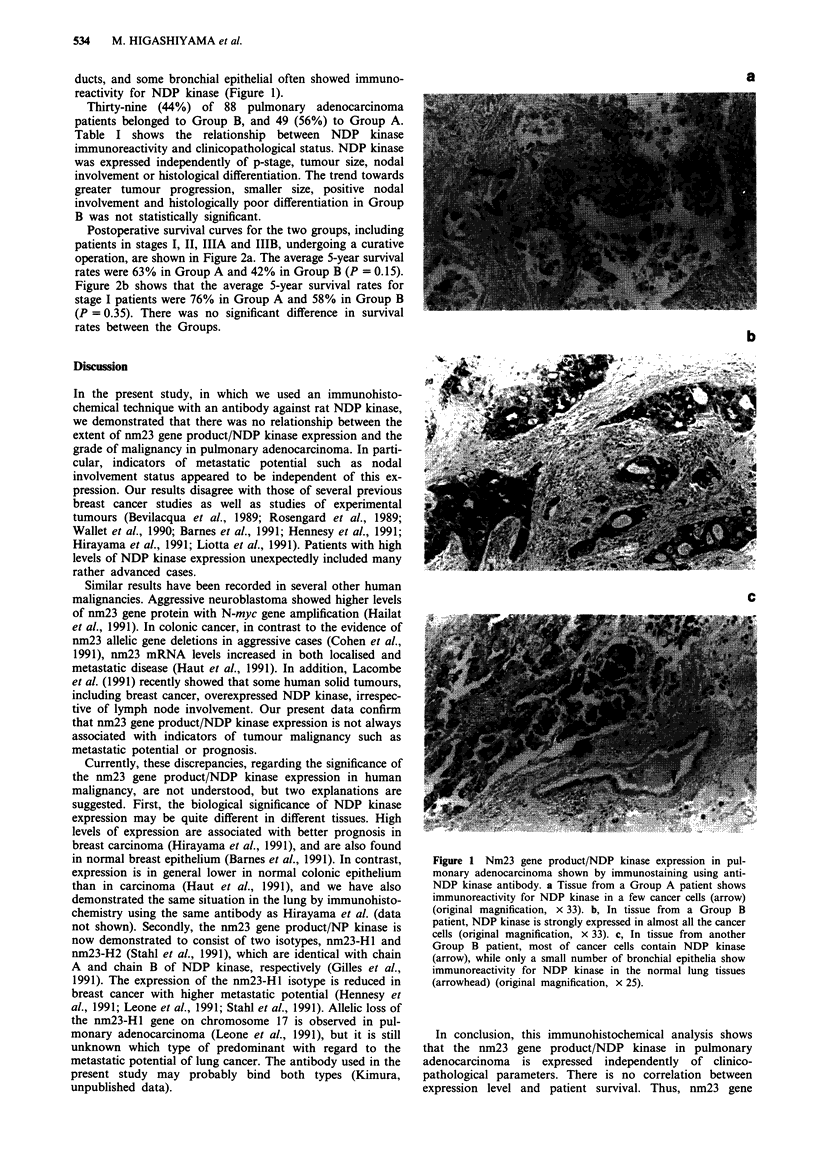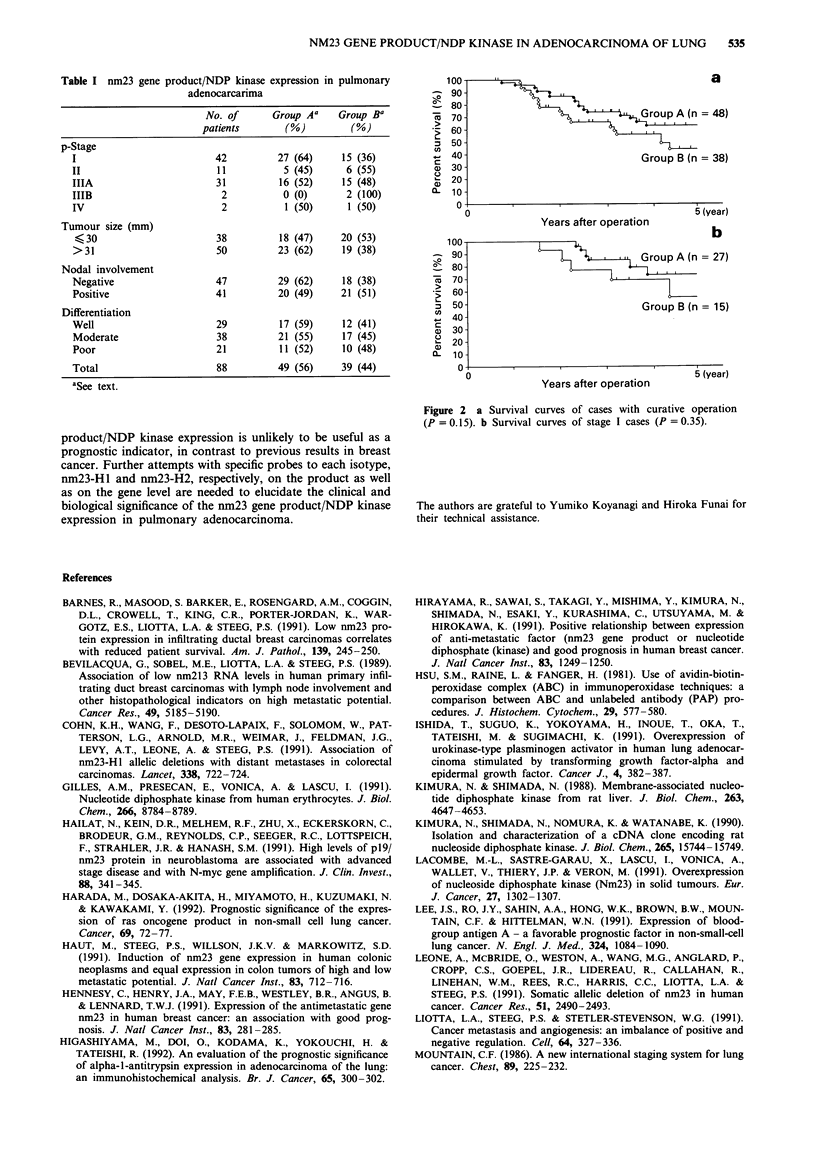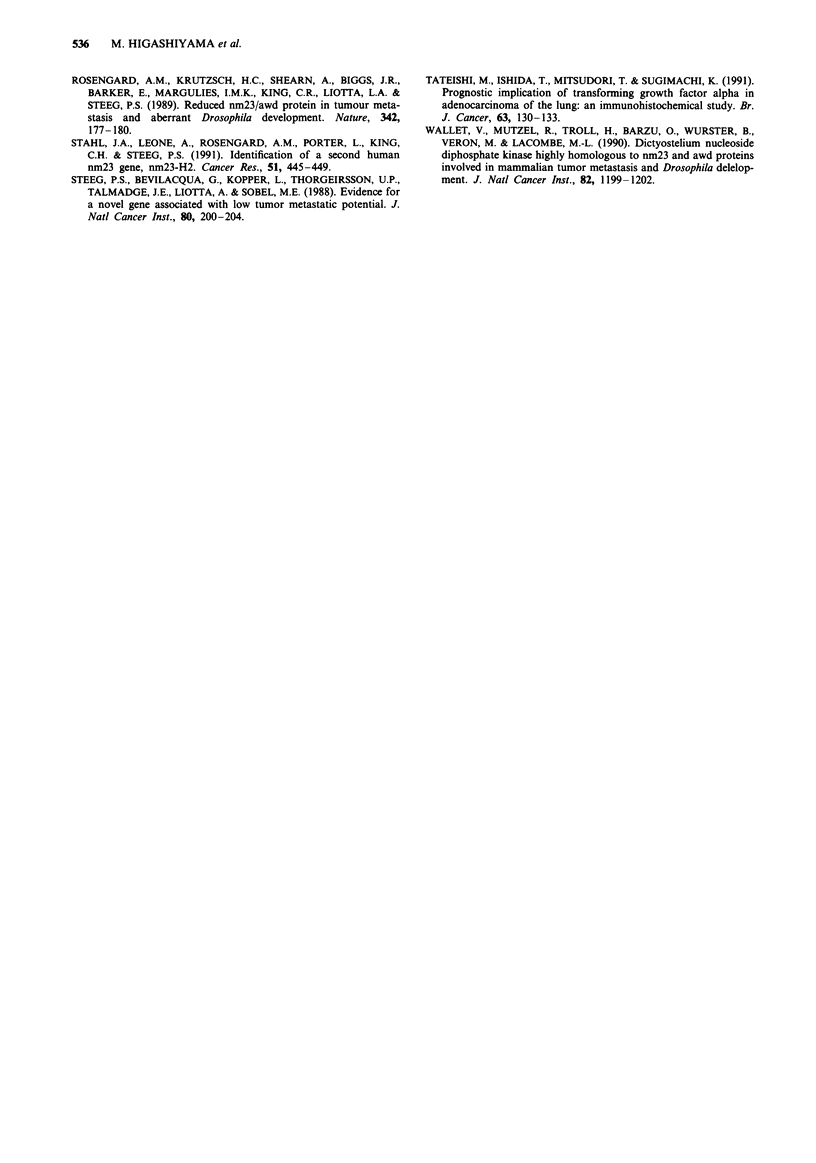# Immunohistochemical analysis of nm23 gene product/NDP kinase expression in pulmonary adenocarcinoma: lack of prognostic value.

**DOI:** 10.1038/bjc.1992.308

**Published:** 1992-09

**Authors:** M. Higashiyama, O. Doi, H. Yokouchi, K. Kodama, S. Nakamori, R. Tateishi, N. Kimura

**Affiliations:** Department of Surgery, Center for Adult Diseases, Osaka, Japan.

## Abstract

**Images:**


Br. J. Cancer (1992), 66, 533–536 © Macmillan Press Ltd., 1992

### Immunohistochemical analysis of nm23 gene product/NDP kinase expression in pulmonary adenocarcinoma: lack of prognostic value

M. Higashiyama[1], O. Doi[1], H. Yokouchi[1], K. Kodama[1], S. Nakamori[1], R. Tateishi[2] & N. Kimura[3]

[1]*Department of Surgery, The Center for Adult Diseases, Osaka,* [2]*Department of Pathology, The Center for Adult Diseases, Osaka,* [3]*Department of Molecular Biology, Tokyo Metropolitan Institute of Gerontology, Tokyo, Japan.*

**Summary** Levels of nm23 gene product/nucleoside diphosphate kinase (NDP kinase) expression have been demonstrated to correlate inversely with metastatic potential in several tumours, indicating that this could be a useful tool as a prognostic indicator. Using an antibody to NDP kinase, levels of nm23 gene product/NDP kinase expression in pulmonary adenocarcinoma were examined immunohistochemically. Of 88 patients tested, 39 (44%; Group B) showed strong immunoreactivity for NDP kinase in most of cancer cells within the tumour tissues, while 49 (56%; Group A) contained few or no NDP kinase-positive cancer cells. Nm23 gene product/NDP kinase was expressed independently of clinicopathological factors, and unexpectedly, no correlation of survival rates between both Groups could be demonstrated. Thus, in pulmonary adenocarcinoma, levels of nm23 gene product/NDP kinase expression may lack prognostic value.

Accurate prediction of the malignant potential of lung cancer, reflecting biological features, such as progression, invasiveness, and metastasis, is an important goal in clinical oncology. In lung cancer, however, and adenocarcinoma in particular, there are no reliable prognostic markers reported for immunohistochemical use, although several candidates have been recently, including oncogene products (Harada *et al.*, 1992), growth factors (Tateishi *et al.*, 1991), proteinases (Ishida *et al.*, 1991), antiproteinases (Higashiyama *et al.*, 1992), and blood type expression (Lee *et al.*, 1991).

It has recently been recognised that the nm23 gene and its product are closely related to the metastatic potential of some tumour cells (Steeg *et al.*, 1988; Liotta *et al.*, 1991). Low levels of nm23 mRNA and the corresponding protein have been reported to reflect high metastatic potential in both experimental animal tumours and human breast cancer (Steeg *et al.*, 1988; Bevilacqua *et al.*, 1989; Rosengard *et al.*, 1989; Barnes *et al.*, 1991; Liotta *et al.*, 1991; Hennesy *et al.*, 1991). The expression of nucleoside diphosphate kinase (NDP kinase), which is now known to be identical to the nm23 gene product (Kimura & Shimada, 1988; Rosengard *et al.*, 1989; Kimura *et al.*, 1990; Wallet *et al.*, 1990), has been investigated in breast cancer by immunohistochemistry: the results suggest that the nm23 gene product/NDP kinase expression may possibly be a useful prognostic marker (Hirayama *et al.*, 1991). Leone *et al.* (1991) first reported somatic allelic deletion of the nm23 gene in pulmonary adenocarcinoma, but to our knowledge, studies of the product level of the nm23 gene have not yet been carried out in lung cancer. We therefore performed immunohistochemical analysis of nm23 product/NDP kinase expression in pulmonary adenocarcinoma to determine its prognostic value.

### Materials and methods

Formalin-fixed and paraffin-embedded tissue blocks from 88 surgically resected specimens of pulmonary adenocarcinoma in our institute, which were well-preserved for immunohistochemical study were examined. All patients except those in stage IV (two patients) due to pulmonary metastasis underwent operations, which seemed curative at the time of operation. Of the 88 patients, 55 were men and 33 were women; their ages ranged from 19 to 78 (mean 61.6). According to the international TNM staging system (Mountain, 1986), 42 of the patients were in pathological stage I (p-stage I), 11 were in pathological stage II (p-stage II), 31 were in pathological stage IIIA (p-stage IIIA), two were in pathological stage IIIB (p-stage IV). With regard to histological degree of differentiation, 29 cases were well differentiated, 38 were moderately differentiated, and 21 were poorly differentiated.

Immunohistochemistry was performed according to a modification of the method of Hsu *et al.* (1981). Briefly, sections (4 µm thick) from each tissue block were deparaffinised, and endogenous peroxidase activity was blocked using 0.3% hydrogen peroxide in methanol. After treatment in 2% normal goat serum, they were incubated with specific antibody to NDP kinase (diluted 1:300), raised against the NDP kinase from rat liver (Kimura & Shimada, 1988). The specificity of the antibody had been confirmed previously by immunoelectrophoretic blotting (Kimura & Shimada, 1988), and this antibody is known to be suitable for immunohistochemical applications (Hirayama *et al.*, 1991). Sections were treated with biotinylated goat anti-rabbit IgG (Vector), and subsequently with avidin-biotin peroxidase complex (Vectastatin ABC kit, Vector).

The peroxidase reaction used 0.02%, 3,3′-diaminobenzidine tetrahydrochloride in 0.05 M TRIS buffer, pH 7.6, containing 0.01% hydrogen peroxide. Sections were counterstained with Mayer's hematoxylin.

Immunostaining results were assessed semi-quantitatively by two of the authors, taking into account the percentage of NDP kinase-positive cancer cells within maximum cut-surface specimens of the tumour tissues, including the surrounding non-cancerous lung tissues. The patients were classified into two groups: Group A patients had less than 30% NDP kinase-positive cancer cells, whilst those in Group B had more than 30%. The chi-quare test was sued for statistical analysis. The Kaplan-Meier method was used to calculate postoperative survival rate, and prognostic significance was evaluated by the generalised Wilcoxon test.

### Results

In this immunohistochemical study using formalin-fixed and paraffin embedded samples, NDP kinase expression was observed in the cytoplasm of pulmonary adenocarcinoma cells with patchy, or often, diffuse staining pattern. Non-cancerous parts of the lung contained no or little NDP kinase with some exceptions: bronchial serous glands, their

Correspondence: M. Higashiyama, Department of Thoracic Surgery, The Center for Adult Diseases, Osaka, Nakamich 1-3-3, Higashinari-ku, Osaka, 537, Japan.
Received 7 February 1992; and in revised form 6 May 1992.

ducts, and some bronchial epithelial often showed immunoreactivity for NDP kinase (Figure 1).

Thirty-nine (44%) of 88 pulmonary adenocarcinoma patients belonged to Group B, and 49 (56%) to Group A. Table I shows the relationship between NDP kinase immunoreactivity and clinicopathological status. NDP kinase was expressed independently of p-stage, tumour size, nodal involvement or histological differentiation. The trend towards greater tumour progression, smaller size, positive nodal involvement and histologically poor differentiation in Group B was not statistically significant.

Postoperative survival curves for the two groups, including patients in stages I, II, IIIA and IIIB, undergoing a curative operation, are shown in Figure 2a. The average 5-year survival rates were 63% in Group A and 42% in Group B ($P = 0.15$). Figure 2b shows that the average 5-year survival rates for stage I patients were 76% in Group A and 58% in Group B ($P = 0.35$). There was no significant difference in survival rates between the Groups.

### Discussion

In the present study, in which we used an immunohistochemical technique with an antibody against rat NDP kinase, we demonstrated that there was no relationship between the extent of nm23 gene product/NDP kinase expression and the grade of malignancy in pulmonary adenocarcinoma. In particular, indicators of metastatic potential such as nodal involvement status appeared to be independent of this expression. Our results disagree with those of several previous breast cancer studies as well as studies of experimental tumours (Bevilacqua *et al.*, 1989; Rosengard *et al.*, 1989; Wallet *et al.*, 1990; Barnes *et al.*, 1991; Hennesy *et al.*, 1991; Hirayama *et al.*, 1991; Liotta *et al.*, 1991). Patients with high levels of NDP kinase expression unexpectedly included many rather advanced cases.

Similar results have been recorded in several other human malignancies. Aggressive neuroblastoma showed higher levels of nm23 gene protein with N-*myc* gene amplification (Hailat *et al.*, 1991). In colonic cancer, in contrast to the evidence of nm23 allelic gene deletions in aggressive cases (Cohen *et al.*, 1991), nm23 mRNA levels increased in both localised and metastatic disease (Haut *et al.*, 1991). In addition, Lacombe *et al.* (1991) recently showed that some human solid tumours, including breast cancer, overexpressed NDP kinase, irrespective of lymph node involvement. Our present data confirm that nm23 gene product/NDP kinase expression is not always associated with indicators of tumour malignancy such as metastatic potential or prognosis.

Currently, these discrepancies, regarding the significance of the nm23 gene product/NDP kinase expression in human malignancy, are not understood, but two explanations are suggested. First, the biological significance of NDP kinase expression may be quite different in different tissues. High levels of expression are associated with better prognosis in breast carcinoma (Hirayama *et al.*, 1991), and are also found in normal breast epithelium (Barnes *et al.*, 1991). In contrast, expression is in general lower in normal colonic epithelium than in carcinoma (Haut *et al.*, 1991), and we have also demonstrated the same situation in the lung by immunohistochemistry using the same antibody as Hirayama *et al.* (data not shown). Secondly, the nm23 gene product/NP kinase is now demonstrated to consist of two isotypes, nm23-H1 and nm23-H2 (Stahl *et al.*, 1991), which are identical with chain A and chain B of NDP kinase, respectively (Gilles *et al.*, 1991). The expression of the nm23-H1 isotype is reduced in breast cancer with higher metastatic potential (Hennesy *et al.*, 1991; Leone *et al.*, 1991; Stahl *et al.*, 1991). Allelic loss of the nm23-H1 gene on chromosome 17 is observed in pulmonary adenocarcinoma (Leone *et al.*, 1991), but it is still unknown which type of predominant with regard to the metastatic potential of lung cancer. The antibody used in the present study may probably bind both types (Kimura, unpublished data).

**Figure 1** Nm23 gene product/NDP kinase expression in pulmonary adenocarcinoma shown by immunostaining using anti-NDP kinase antibody. **a** Tissue from a Group A patient shows immunoreactivity for NDP kinase in a few cancer cells (arrow) (original magnification, × 33). **b**, In tissue from a Group B patient, NDP kinase is strongly expressed in almost all the cancer cells (original magnification, × 33). **c**, In tissue from another Group B patient, most of cancer cells contain NDP kinase (arrow), while only a small number of bronchial epithelia show immunoreactivity for NDP kinase in the normal lung tissues (arrowhead) (original magnification, × 25).

In conclusion, this immunohistochemical analysis shows that the nm23 gene product/NDP kinase in pulmonary adenocarcinoma is expressed independently of clinicopathological parameters. There is no correlation between expression level and patient survival. Thus, nm23 gene

**Table I** nm23 gene product/NDP kinase expression in pulmonary adenocarcarima

| | *No. of patients* | *Group A*[a] *(%)* | *Group B*[a] *(%)* |
|---|---|---|---|
| p-Stage | | | |
| I | 42 | 27 (64) | 15 (36) |
| II | 11 | 5 (45) | 6 (55) |
| IIIA | 31 | 16 (52) | 15 (48) |
| IIIB | 2 | 0 (0) | 2 (100) |
| IV | 2 | 1 (50) | 1 (50) |
| Tumour size (mm) | | | |
| ≤30 | 38 | 18 (47) | 20 (53) |
| >31 | 50 | 23 (62) | 19 (38) |
| Nodal involvement | | | |
| Negative | 47 | 29 (62) | 18 (38) |
| Positive | 41 | 20 (49) | 21 (51) |
| Differentiation | | | |
| Well | 29 | 17 (59) | 12 (41) |
| Moderate | 38 | 21 (55) | 17 (45) |
| Poor | 21 | 11 (52) | 10 (48) |
| Total | 88 | 49 (56) | 39 (44) |

[a]See text.

product/NDP kinase expression is unlikely to be useful as a prognostic indicator, in contrast to previous results in breast cancer. Further attempts with specific probes to each isotype, nm23-H1 and nm23-H2, respectively, on the product as well as on the gene level are needed to elucidate the clinical and biological significance of the nm23 gene product/NDP kinase expression in pulmonary adenocarcinoma.

**Figure 2** **a** Survival curves of cases with curative operation ($P$ = 0.15). **b** Survival curves of stage I cases ($P$ = 0.35).

The authors are grateful to Yumiko Koyanagi and Hiroka Funai for their technical assistance.

### References

BARNES, R., MASOOD, S. BARKER, E., ROSENGARD, A.M., COGGIN, D.L., CROWELL, T., KING, C.R., PORTER-JORDAN, K., WARGOTZ, E.S., LIOTTA, L.A. & STEEG, P.S. (1991). Low nm23 protein expression in infiltrating ductal breast carcinomas correlates with reduced patient survival. *Am. J. Pathol.*, **139**, 245–250.

BEVILACQUA, G., SOBEL, M.E., LIOTTA, L.A. & STEEG, P.S. (1989). Association of low nm213 RNA levels in human primary infiltrating duct breast carcinomas with lymph node involvement and other histopathological indicators on high metastatic potential. *Cancer Res.*, **49**, 5185–5190.

COHN, K.H., WANG, F., DESOTO-LAPAIX, F., SOLOMOM, W., PATTERSON, L.G., ARNOLD, M.R., WEIMAR, J., FELDMAN, J.G., LEVY, A.T., LEONE, A. & STEEG, P.S. (1991). Association of nm23-H1 allelic deletions with distant metastases in colorectal carcinomas. *Lancet*, **338**, 722–724.

GILLES, A.M., PRESECAN, E., VONICA, A. & LASCU, I. (1991). Nucleotide diphosphate kinase from human erythrocytes. *J. Biol. Chem.*, **266**, 8784–8789.

HAILAT, N., KEIN, D.R., MELHEM, R.F., ZHU, X., ECKERSKORN, C., BRODEUR, G.M., REYNOLDS, C.P., SEEGER, R.C., LOTTSPEICH, F., STRAHLER, J.R. & HANASH, S.M. (1991). High levels of p19/nm23 protein in neuroblastoma are associated with advanced stage disease and with N-myc gene amplification. *J. Clin. Invest.*, **88**, 341–345.

HARADA, M., DOSAKA-AKITA, H., MIYAMOTO, H., KUZUMAKI, N. & KAWAKAMI, Y. (1992). Prognostic significance of the expression of ras oncogene product in non-small cell lung cancer. *Cancer*, **69**, 72–77.

HAUT, M., STEEG, P.S., WILLSON, J.K.V. & MARKOWITZ, S.D. (1991). Induction of nm23 gene expression in human colonic neoplasms and equal expression in colon tumors of high and low metastatic potential. *J. Natl Cancer Inst.*, **83**, 712–716.

HENNESY, C., HENRY, J.A., MAY, F.E.B., WESTLEY, B.R., ANGUS, B. & LENNARD, T.W.J. (1991). Expression of the antimetastatic gene nm23 in human breast cancer: an association with good prognosis. *J. Natl Cancer Inst.*, **83**, 281–285.

HIGASHIYAMA, M., DOI, O., KODAMA, K., YOKOUCHI, H. & TATEISHI, R. (1992). An evaluation of the prognostic significance of alpha-1-antitrypsin expression in adenocarcinoma of the lung: an immunohistochemical analysis. *Br. J. Cancer*, **65**, 300–302.

HIRAYAMA, R., SAWAI, S., TAKAGI, Y., MISHIMA, Y., KIMURA, N., SHIMADA, N., ESAKI, Y., KURASHIMA, C., UTSUYAMA, M. & HIROKAWA, K. (1991). Positive relationship between expression of anti-metastatic factor (nm23 gene product or nucleotide diphosphate (kinase) and good prognosis in human breast cancer. *J. Natl Cancer Inst.*, **83**, 1249–1250.

HSU, S.M., RAINE, L. & FANGER, H. (1981). Use of avidin-biotin-peroxidase complex (ABC) in immunoperoxidase techniques: a comparison between ABC and unlabeled antibody (PAP) procedures. *J. Histochem. Cytochem.*, **29**, 577–580.

ISHIDA, T., SUGUO, K., YOKOYAMA, H., INOUE, T., OKA, T., TATEISHI, M. & SUGIMACHI, K. (1991). Overexpression of urokinase-type plasminogen activator in human lung adenocarcinoma stimulated by transforming growth factor-alpha and epidermal growth factor. *Cancer J.*, **4**, 382–387.

KIMURA, N. & SHIMADA, N. (1988). Membrane-associated nucleotide diphosphate kinase from rat liver. *J. Biol. Chem.*, **263**, 4647–4653.

KIMURA, N., SHIMADA, N., NOMURA, K. & WATANABE, K. (1990). Isolation and characterization of a cDNA clone encoding rat nucleoside diphosphate kinase. *J. Biol. Chem.*, **265**, 15744–15749.

LACOMBE, M.-L., SASTRE-GARAU, X., LASCU, I., VONICA, A., WALLET, V., THIERY, J.P. & VERON, M. (1991). Overexpression of nucleoside diphosphate kinase (Nm23) in solid tumours. *Eur. J. Cancer*, **27**, 1302–1307.

LEE, J.S., RO, J.Y., SAHIN, A.A., HONG, W.K., BROWN, B.W., MOUNTAIN, C.F. & HITTELMAN, W.N. (1991). Expression of blood-group antigen A – a favorable prognostic factor in non-small-cell lung cancer. *N. Engl. J. Med.*, **324**, 1084–1090.

LEONE, A., McBRIDE, O., WESTON, A., WANG, M.G., ANGLARD, P., CROPP, C.S., GOEPEL, J.R., LIDEREAU, R., CALLAHAN, R., LINEHAN, W.M., REES, R.C., HARRIS, C.C., LIOTTA, L.A. & STEEG, P.S. (1991). Somatic allelic deletion of nm23 in human cancer. *Cancer Res.*, **51**, 2490–2493.

LIOTTA, L.A., STEEG, P.S. & STETLER-STEVENSON, W.G. (1991). Cancer metastasis and angiogenesis: an imbalance of positive and negative regulation. *Cell*, **64**, 327–336.

MOUNTAIN, C.F. (1986). A new international staging system for lung cancer. *Chest*, **89**, 225–232.

ROSENGARD, A.M., KRUTZSCH, H.C., SHEARN, A., BIGGS, J.R., BARKER, E., MARGULIES, I.M.K., KING, C.R., LIOTTA, L.A. & STEEG, P.S. (1989). Reduced nm23/awd protein in tumour metastasis and aberrant *Drosophila* development. *Nature*, **342**, 177-180.

STAHL, J.A., LEONE, A., ROSENGARD, A.M., PORTER, L., KING, C.H. & STEEG, P.S. (1991). Identification of a second human nm23 gene, nm23-H2. *Cancer Res.*, **51**, 445-449.

STEEG, P.S., BEVILACQUA, G., KOPPER, L., THORGEIRSSON, U.P., TALMADGE, J.E., LIOTTA, A. & SOBEL, M.E. (1988). Evidence for a novel gene associated with low tumor metastatic potential. *J. Natl Cancer Inst.*, **80**, 200-204.

TATEISHI, M., ISHIDA, T., MITSUDORI, T. & SUGIMACHI, K. (1991). Prognostic implication of transforming growth factor alpha in adenocarcinoma of the lung: an immunohistochemical study. *Br. J. Cancer*, **63**, 130-133.

WALLET, V., MUTZEL, R., TROLL, H., BARZU, O., WURSTER, B., VERON, M. & LACOMBE, M.-L. (1990). Dictyostelium nucleoside diphosphate kinase highly homologous to nm23 and awd proteins involved in mammalian tumor metastasis and *Drosophila* delelopment. *J. Natl Cancer Inst.*, **82**, 1199-1202.